# Enacting person‐centredness in integrated care: A qualitative study of practice and perspectives within multidisciplinary groups in the care of older people

**DOI:** 10.1111/hex.12803

**Published:** 2018-07-13

**Authors:** Lisa K. Riste, Peter A. Coventry, Siobhan T. Reilly, Peter Bower, Caroline Sanders

**Affiliations:** ^1^ NIHR School for Primary Care Research University of Manchester Manchester UK; ^2^ Department of Health Sciences University of York York UK; ^3^ Division of Health Research Lancaster University Lancaster UK

**Keywords:** integrated care, multidisciplinary groups, older people, person‐centredness

## Abstract

**Background:**

Person‐centredness is important in delivering care for long‐term conditions. New models of care aim to co‐ordinate care through integration of health and social care which require new ways of working, often remotely from the patient.

**Objective:**

To describe how person‐centred care is enacted within multidisciplinary groups (MDGs) created as part of a new service, integrating health and social care for older people.

**Methods:**

We followed the implementation of eight neighbourhood MDGs, observing and interviewing staff from three MDGs at different phases of programme implementation using semi‐structured topic guides.

**Results:**

Thirty‐four MDG meetings were observed and 32 staff interviewed. Three core themes were identified which impacted on enactment of person‐centred care: the structural context of MDGs enabling person‐centred care; interaction of staff and knowledge sharing during the MDG meetings; and direct staff involvement of the person outside the MDG discussion.

**Conclusions:**

This study provides new insights into attempts to enact person‐centred care within a new model of service delivery. Teams did what they could to enact person‐centred care in the absence of the “real” patient within MDG meetings. They were successful in delivering and co‐ordinating some aspects of care (eg prompting medication reviews, referring to social worker, health improvement and arranging further multidisciplinary team meetings for complex cases). This “absence of patients” and time pressures within the MDGs led to reliance on the “virtual” record, enhanced by additional “soft” knowledge provided by staff, rather than ensuring the patient's voice was included.

## 1 INTRODUCTION

In 2012, people aged 65 +  accounted for 17% of the population in England, but represented 54% of hospital bed days.^1^ Many policies emphasize the importance of caring for older people for as long as possible at home,^2^ to reduce costly hospital admissions. Ageing populations experience multimorbidity, but often receive care from numerous professionals spanning primary, secondary and social care. Patients often cite lack of communication between such services,^3^ especially at the interface between services, and describe “falling between the gaps.”^4^

### 1.1 Integrated care and case management

Integrated care is often proposed as a way of better managing older patients with significant health and social care needs. Consensus on the meaning of “integrated care” is difficult,^5^ but many definitions focus on bringing together health and social care professionals involved in care across hospital and community settings. This “*patient level integration*” can help older people navigate complex care systems and reduce inappropriate care use,^6^ often through a *case management* approach.

Case management is a strategy for organizing services for an individual patient^7^ and is increasingly used as a mechanism to integrate services, with an expectation it will reduce hospital admissions.^8^, ^9^, ^10^ Case management generally involves (i) *care planning* to assess a persons’ needs and (ii) *care co‐ordination,* both between those offering services, and the person receiving them, usually via a key worker.

### 1.2 Person‐centred care in case management

Person‐centred care encourages clinicians to “see the person behind the patient.”^11^ It seeks to provide a more holistic approach to providing care people will want and use, by incorporating knowledge about them and their family, rather than treating a specific disease or condition. This lead to person‐centred care being included as one of four standards in the National Services Framework for Older People.^12^

The NHS “National Collaboration for Integrated Care and Support”^13^ adopted a person‐centred definition focussed on patient experience. For example, they suggested that patients receiving integrated care might be more likely to report that “*I can plan my care with people who work together to understand me and my carer(s), allow me control, and bring together services to achieve the outcomes important to me*.” Integrated services are often associated with patient experience of services that are “joined up” and centred around the individual patient.

Much of the literature around delivering patient/person‐centred care arose in the context of long‐term relationships between single practitioners and patients,^14^ which may not be generalizable to models of care such as case management which are more reliant on decision making outside of face‐to‐face encounters, and involve large multidisciplinary teams (MDTs). A literature review and evidence synthesis identified three core themes of patient‐centred care: patient participation and involvement; the relationship between the patient and the health‐care professional, and the context where their care is delivered (including access, organizational systems and the therapeutic environment).^15^ Ideally, patients are presented with all potential options around their management and then participate in shared decision making with professionals.

Although there is a link between integrated and person‐centred care, achieving both is not without challenge. Core features of person‐centred care include detailed knowledge of the patient and an effective “therapeutic relationship,” which may be more difficult to achieve in team‐based care, as a patient seeing a variety of professionals may feel less able to build up a rapport.^16^ There may also be tensions between the aims of integrated and person‐centred care. The latter is designed to ensure that care is “closely congruent with, and responsive to patients” wants, needs and preferences,^17^ whereas a major impetus behind the promotion of integrated care is to reduce hospital admissions and costs, and to do so across a defined populations of “at‐risk” patients which may involve significant numbers of individuals. Greenfield et al explored patient narratives in integrated care and describe a continuum of experience between having “space” to be heard and seen vs feeling “translucent,” “unseen,” and “unheard.”^18^ They highlighted that person‐centredness based on relationship dyads may not correspond to the reality of working in integrated care settings and suggested that patient experience of compassion was often absent.

There has been little research into how person‐centred care is achieved in integrated MDTs using case management. Previous ethnographic studies have offered valuable insights into how contradictory organizational pressures are managed within MDTs.^19^ In the context of care pathways, Allen found that redesign reduced person‐centredness with a tendency for people to be “ushered” down a particular route often with little consultation.

We adopted a similar methodology to Allen,^19^ using qualitative interviews and detailed observations to reflect critically on the possibility of enabling person‐centred care in the context of multidisciplinary case discussions and team‐based decision making when the person is presented as a “case,” but is not present.

## 2 METHODS

### 2.1 Context of the study—the Integrated Care Programme

The Integrated Care Programme (ICP) was a large‐scale transformation of services for patients over 65 years of age, including significant integration across organizations and services in an area in the north‐west of England.^20^ Budgets for older people were pooled in order to drive the implementation of ICP.

Public engagement during the development phase of the ICP included public governors, patient organizations, and older peoples’ forums and reference groups. Real data were used to create a fictitious character *Sally Ford*, helping staff focus on improving the experience of older people similar to “Mrs Smith,” who illustrated older people's issues in Torbay.^21^

As part of the ICP,^22^ the population was stratified into levels of need. Multidisciplinary groups (MDGs) were set up to provide integrated case management for the 3100 people identified receiving 3 or more visits per week from district nursing and/or social care.

Neighbourhood MDGs operated fortnightly and included a project manager supported by an administrator. Each MDG included a GP and/or practice nurse from between 4 and 10 local GP practices. Nursing and social care practitioners cochaired meetings which were also attended by a geriatrician, mental health advance practitioner and health improvement officer. A shared care record (SCR) was created which was populated with data from existing primary care databases, with additional data being input prior to meetings. The SCR was displayed at MDG meetings during patient discussions and updated with outcomes and actions agreed. These actions included referrals to social care, mental health or health improvement with additional meetings held for some of the most complex cases. The professional best known to the patient was usually assigned the role of care co‐ordinator within the SCR.

### 2.2 Data collection

Our study used multiple qualitative methods including observations and interviews to research the work conducted and to explore perspectives of MDG team members. This provided insights into staff expectations, how MDGs worked in practice, and staff perceptions of being able to deliver person‐centred care. Neighbourhoods chosen for in‐depth observations included one from each of three phases of implementation of the ICP. Selection ensured those with unique staff roles within its MDGs were included (neighbourhood engagement manager and member of the hospital rehabilitation team). Ethical approval was granted by National Research Ethics Service Committee North West—Lancaster (NRES 14/NW/0206).

Thirty‐four MDG meetings were observed (March—December 2015), covering all eight neighbourhoods covered by the ICP, implemented in three waves (two pilot sites in March 2014, with later phases in January and March 2015). Sequential observations and extensive field notes were taken allowing potential follow‐up of cases discussed.

Other meetings outside the MDGs were observed to understand underlying processes, for example social care and nursing pre‐MDG meetings, demonstrations of SCR creation and data population, cochair meetings to ensure co‐linearity across the neighbourhoods. ICP documentation was used to compare the operational aspirations of the MDGs with emergent findings.

Semi‐structured interviews were conducted with 27 staff attending MDGs, covering all roles (Table 1), plus 5 others whose work supported the MDGs. Interviews were conducted by a research fellow (LR) trained in qualitative methods and averaged 59 (range 34‐96) minutes. The topic guide covered roles and responsibilities, patient interaction and project progress. Since this *article* focuses on how MDG staff enacted person‐centeredness, interviews with patients and carers are reported elsewhere.^22^

**Table 1:** Summary of person‐centred care enabler “themes” by MDG role

MDG role n interviews	MDG structure Roles and records in collating data	MDG meetings Enabling sharing “soft” knowledge	Making time Direct patient interactions
2 Social Care A/P (cochairs)	Input into SCR and own CareFirst system	Input from social care team to A/P cochair	Time pressure restricts joint assessment
5 A/P Nurse (cochairs)	Create and input SCR, time‐consuming but necessary	Input from district nursing team to A/P cochair	Frustration: need joint holistic assessment visits
2 General Practice Nurses	Access issues—input via MDG cochair	Check/(re)order blood tests/Share good practice	Consulting patients and offering choice
6 General Practitioners	Access issues—input via MDG cochair	Prioritizing patients and incorporating family views	Limited to discussion during consultations
2 Geriatricians	Importance of baseline status in assessing change	Discuss preventive measures and minimizing unwanted interventions	Medication reviews to reduce polypharmacy, prevent falls
1 Mental Health A/P	Limited involvement—IT system compatibility	Paper summary used in meetings	Referral for assessment or to mental health MDT
a1 Rehabilitation service	Use of SCR in hospital context	Attendance limited by time	Discussion pre‐MDG and home visit pre‐discharge
a1 Intermediate care	Frustrated at entry on SCR and own CareFirst system	Provided patient experience and update on intermediate care bed availability	—
a1 Neighbourhood Manager	SCR use for reviewing recent hospital discharges	Feedback knowledge, collate local service directory	Phone patients helping prevent hospital readmission
3 Health Improvement Officers	No access	Try to match patient needs to interventions	Phone and visit to discuss and offer interventions
2 MDG Project Managers	SCR set‐up, numbers indicate MDG progress	—	—
1 MDG Administrator	Access SCR and liaise with GPs to prioritize patients	Some information provision from previous role	Important to involve patient and families

A/P, advance practitioner; MDG, multidisciplinary group; MDT, multidisciplinary team; SCR, shared care record.

a Only present in one MDG, limited availability.

Participants provided written consent and interviews were digitally audiotaped and transcribed. Interview transcripts were verified against the audio file, anonymized and re‐read ahead of initial coding. Transcripts, field notes and supporting documents were organized using NVivo v10.^23^ A thematic analysis was conducted, drawing upon some techniques of a grounded theory approach, including a constant comparative technique whereby iterative analysis informed adaptation of the topic guides and further sampling. Initial coding treated interviews and observation notes independently, with memos enabling deeper insights as the project progressed. The qualitative project team met monthly to review emerging themes and to agree themes and subthemes.

## 3 RESULTS

Local groups involved during the consultation phase of the ICP recognized the importance of direct consultation with older people about their involvement in this new model of care.

We started out by actually engaging older people and asking them what was important to them…..[we] got them to try and understand what integrated care and Sally Ford [was]…..in a language they could understand. (Charity representative, ID26)

Observations and interviews, however, revealed that patients and/or carers were not invited to participate in MDG meetings and very few were routinely involved in discussions beforehand. Despite the absence of the patient at the MDG meetings, staff attempted to deliver person‐centred care in a variety of ways.

We have therefore structured the results into three core themes concerning the enactment of person‐centred care, representing a continuum of increasing levels of patient involvement. Table 1 shows a summary of these themes, differentiated by the various staff involved in the MDG meetings, and we provide direct interview extracts below.

### 3.1 Roles and records: MDG structures supporting person‐centred care

Integration in the ICP was mirrored in MDGs, with nursing and social care leads cochairing meetings. Input from general practice was usually by a nominated GP, sometimes accompanied by or represented by a practice nurse. Acute or community geriatricians also attended. Mental health was represented by an advanced practitioner, and in two neighbourhoods, a consultant psychiatrist. Health improvement officers helped link older people into activities in their communities such as befriending schemes and exercise classes.

GPs felt the multidisciplinary approach increased the solutions available, but emphasized patient choice was important in meeting their needs.

…..For the patients hopefully we're optimising what services are available and giving them options as to what they want and addressing as many needs as we can, ……so encouraging more multidisciplinary people into dealing with patients’ problems means that hopefully they've got different angles of solving them. (GP, ID21)

The SCR was viewed as an important structural support for person‐centred care. Although utilizing entries from existing patient records, there were numerous systems in place which did not directly interact with each other. Nursing and social care leads created and populated the SCR, and recognized its importance in being able to pool information from different sources whilst helping avoid patients having to repeat information.

…..I worked in hospital, they get asked lots of questions in A&E, then they move to the ward, they get asked another lot of questions, then the social worker might come to see them and they get… But by looking at that [shared care] record you should be able to see that they've got carers or that they have the district nurse and whatever, and just clarify with them rather than getting them to repeat all over again six times over. (MDG nurse, ID7)

Although MDG nurses recognized the importance of the SCR work, there was a perception that this work was not valued by non‐MDG nursing colleagues.

…when I input [SCRs] on the iPad, I used to sit out in the carpark because you can still get Wi‐Fi there, but nobody can see you. But it's almost like a dirty little secret doing admin when you're a nurse, because you should be attending to patients….. (MDG nurse, ID5)

### 3.2 MDG meetings: enabling sharing “soft” knowledge

In addition to MDG professionals being able to optimize patient care by conducting medication reviews, making referrals etc., less concrete interactions also occurred.

This second theme reflects how staff viewed the MDG meetings as an opportunity to share “soft” knowledge, that is knowledge extending beyond the “usual” medical diagnoses or treatments captured by the SCR as “data.” This “soft” knowledge is generally unspoken and unwritten but may provide meaning and context to existing routine data.^24^ Although falling short of *shared decision making*, sharing this knowledge of patients and their families helped to provide a person‐centred approach to care planning within the context of the group discussions.

…it's a cliché, but a GP or a medic saying, this is what that person needs to have in terms of care or treatment, and a social worker who might have seen the person in the home saying, well I can tell you the way that this person lives their life that won't happen. (Social Care, ID30)

MDG Nurses and social workers also captured this shared knowledge *within* their teams at neighbourhood “safety huddle” and locality meetings. This collected knowledge was used in care planning and was cited to avoid patients repeating their stories.

Do I need to go to that person's house and speak to them and get it from them or can I get it from their nurse? Can I get it from their social worker? So do I need to see that person? That's the question… (MDG Nurse, ID31)

Longstanding patient‐practitioner relationships in primary care were important sources of this knowledge, exceeding that obtained from in‐depth assessments.

The level of affection that the GPs talk about their patients I think is wonderful ….. they know the families, they know their situations and that makes a difference as well. (Health Improvement Officer, ID10)

….I have knowledge not just of the individuals, but also their extended families. With this experience I can offer information about their support networks. (Practice Nurse, ID15)

…it's been very interesting to realise quite how in depth they [GPs] know their patients, because we don't, we just know them…even by doing the comprehensive geriatric assessment. (Geriatrician, ID29)

An example of this was when a man appeared confused at the accident and emergency department after moving into warden‐supported accommodation. Prone to wandering, his GP and the MDG social care lead discussed using a GPS tracker, after the GP commented he always wore the same coat. This solution could help wardens ensure his safety, whilst avoiding another potentially disruptive accommodation change.

Shared multidisciplinary understanding of patient's lives also helped provide solutions to previously insurmountable issues, such as problems with access to care.

…we were saying about this fellow going at night to [a hospital] appointment, and I was saying about the care agency going in later, the social workers can arrange that; and she said, “but they finish at eight o'clock”; so everything I was saying was being answered really negatively. ….. But then we realised that maybe ….. the evening district nurses could go and put him in bed that night. (MDG Nurse, ID15)

Within the confines of the MDG process, staff recognized knowledge sharing allowed personalization of care, alongside knowledge of the persons’ needs and wishes.

… [person‐centred care] it's about the person knowing what's going on and then having their view heard, et cetera, but being person‐centred by having a number of people sharing what they know it'll hopefully bring a more personalised approach…… you know, for the person's own benefit, but what they want for themselves, what the outcomes of the individual themselves want to do. (Social Care, ID26)

### 3.3 Making time: direct patient interactions

Many quotes from MDG nurses and social care chairs voiced frustration around the divergence from the initial Standardised Operating Procedure^20^ which suggested biopsychosocial assessments should be carried out prior to MDG discussions.

I feel that should be me going to their house and saying, oh, this is what's been identified, is this right, we're going to bring it to this, how do you feel about that and what's important to you. (MDG nurse, ID5)

MDG nurses felt complex patients required joint assessments with social care colleagues to gain a true understanding of the issues people faced, enabling a more person‐centred and realistic approach to care planning. One example provided showed the benefit of a joint assessment.

I went to see somebody with [social care chair], it was one of her clients,… there was a situation going on [patients husband was in hospital so her care package was being reassessed] … She's not known to nurses because I had a look before I went. I thought how is she not known to nurses? She's got equipment coming out of her ears, she's got high risk pressure cushions, she's got a standing hoist, she's got a bed, she's got everything. (MDG Nurse, ID 36)

The patient asked the nurse to look at a sore which wasn't healing despite carers using the cream prescribed. The nurse found a pressure sore and was then able with the patient's consent to refer her to the district nursing pressure care team.

Although difficult to facilitate, some staff felt patients should have the opportunity to attend MDGs if they wished, despite appreciating that discussions outside MDG meetings might be necessary.

….. why wouldn't they [patients] want to be involved? What is being discussed there that the person and their families wouldn't find relevant, appropriate or interesting? (Charity representative, ID26)

As a proxy, existing practitioners who knew the patient best were appointed as care co‐ordinators, linking patients to the MDG.

…ideally the care coordinator would be in place and it would be that person that would then go and have that discussion with them and give them the feedback from the meeting and everything. (Social Care, ID6)

Time pressures limited the care co‐ordinators’ ability to do this. The ability to consult directly with patients was best demonstrated by health improvement officers. With knowledge of locally available services and activities for older people, the health improvement team acted as both provider and broker, matching patient needs to existing activities and groups. Their staff visited or telephoned patients to discuss options and re‐engaged some in community activities.

I spoke to one lady yesterday and I'm hoping that she's going to join our Healthy Lifestyles group and knowing a little bit about her, …..the practice manager put a really good write up on the MDG shared information, really facilitated the conversation with her,….. she felt that people had cared enough about her to share the relevant information. (Health Improvement Officer, ID10)

Another patient's participation was constrained by carer schedule:

…..the timing of the carers didn't fit with the timing of the [exercise] group,…..we'd be able to sit around the table and have that discussion quite quickly and come up with a resolution rather than the toing and froing that had to happen ….. [by then] the group might have finished and the person might not have benefited from participation. (Health Improvement Officer, ID10)

Towards the end of the MDG observation period, a “*Summary of existing risks and issues*” was introduced to the SCR. This more proactive approach to care planning, when combined with direct patient consultation, led to positive outcomes for a small number of patients.

One patient was regularly taken to A&E by his carers with blood in his vomit (due to a pre‐existing condition). The geriatrician had previously advised his GP this was unnecessary. Following MDG discussion the geriatrician liaised directly with the patient creating a care plan, which stated “the patient would advise carers if he needed to go to hospital.” (Fieldnote observation, April 2015)

Even when staff were able to invest time by virtue of their roles, they respected that the choice to engage with any suggestions made ultimately lay with the patient.

…..some people, no matter what they do, you can only try, and I think as long as you know you've tried your very best, then people are entitled to make their own decisions. (Neighbourhood Manager, ID30)

## 4 DISCUSSION

### 4.1 Summary of main findings

This article illustrates how new MDGs set up to integrate primary and secondary health and social care were able to enact some degree of person‐centredness, despite patients being absent from MDG discussions.

Integrated Care Programme documentation^20^ described a named care co‐ordinator consenting patients before discussion at MDG meetings and incorporating their needs and preferences into care planning. Some GPs and practice nurses reported speaking with patients beforehand, and feeding back the outcome of MDG meetings to patients during routine appointments, but workload and service pressures meant there was little time to contact patients ahead of MDG discussions. Whilst care co‐ordinators were nominated, many viewed their roles more as co‐ordinating care between services rather than providing liaison between the patient and the MDG.

The findings of the current study suggest that the practices within participating MDGs were mostly focused on enabling integrated care among services, yet there were aspirations for person‐centred care reflected in the work of MDGs. Observation of discussions within MDG meetings, and interviews with clinicians indicated that their decisions and practices reflected improvements in some aspects of integrated care deemed important by patients such as enabling greater “holism,”^18^ and in the micro aspects of care that enabled small and important changes, akin to Mol's “*tinkering at the edges of care*.”^25^

In some senses, the new structures and tools (such as the SCR) opened up a new space to see the patient in a virtual sense. However, discussions indicated that important biographical information and small details of day‐to‐day care were absent from these records. Such omissions, combined with the absence of patients, posed a barrier to enactment of person‐centred care in practice. The case discussions that took place during MDG meetings helped incorporate this soft “knowledge” or intelligence, providing meaning and context to the SCR, helping construct what Hamilton describes as an “evidential patient.”^26^ Whilst the SCR was a key platform for integrating care, there were tensions between quantity of SCRs completed, and their quality in terms of including sufficient data to aid care co‐ordination and planning.

Although the patients being discussed were identified from a risk profiling exercise based on use of nursing and social care services, GPs were often asked to prioritize patients for discussion. It is likely therefore that those patients discussed were likely to represent more frequent practice attenders. Whilst GPs seemed less aware of patients’ problems where they had not either seen them at home or in the surgery recently, nursing and social care were able to provide this detail. For patients well‐known to these services, the MDG facilitated decision making at a tertiary level, by linking them into local activities such as providing support to attend afternoon tea, reducing their social isolation.

As noted earlier, integrated care through case management is a population strategy, designed to be applied to significant numbers of patients deemed “at risk.” To meet this population approach, rigorous ICP project management targets, combined with health and social care pressures during implementation may have prevented more in‐depth patient involvement in the MDGs. Despite observing one neighbourhood MDG from each of the three roll‐out phases, even MDGs that had been running over 1 year may have not had sufficient time to fully embed the new ways of working. Whether person‐centredness would increase as the pressure to meet targets receded remains an important question.

Greenfield et al^18^ discuss the theoretical differences between models of integrated care and person‐centeredness. They describe integrated care as congruent with a macro and structural view of a complex health‐care system requiring co‐ordination of multiple clinicians and organizational processes. On the other hand, person‐centeredness is congruent with a micro perspective focusing on the interactional level between clinicians and patients. Whilst these concepts can be viewed to have a similar ethos, they have largely operated from differing perspectives, and questions remain as to whether person‐centeredness can be effectively embedded within the practices of integrated care.

### 4.2 Strengths and limitations

Qualitative interviews with MDG staff provided an insight into how they expected person‐centredness to be achieved within the ICP, and this combined with the observations during MDG meetings helped to identify the processes through which it was enacted. Interviews with patients and/or their carers are unlikely to have yielded this level of detail given how few appreciated the changes in how their health and social care was now being delivered.

There were a disproportionate number of interviews with nursing and GPs attending the MDG meetings compared to social care staff, and we were unable to interview the consultant psychiatrist. This reflects the caseload pressures and also the mechanisms by which staff from social care and mental health were involved in the MDGs.

One major limitation was the timing of the observation and interviews. Staff interviews indicated that tensions arose during the implementation of the MDGs, and the focus on project objectives and targets might have led to patients being *less* involved in decisions made about their care rather than *more* involved. Changes observed latterly at MDG meetings suggest a shift towards a greater involvement of patients and carers in the process. The development and use of shared care plans will require patient and carer input and indicate a definite investment in person‐centredness by the MDGs.

### 4.3 Relationship of the findings to the wider literature

Our findings echo those from the North‐West London Integrated Care Pilot for patients aged 75 years or more or with a diagnosis of diabetes,^27^ where MDG staff reported team meetings were more provider—than patient‐oriented. In‐depth analysis of utterances in these multidisciplinary meetings found relatively low levels of “integrative intensity” (defined as communication that is solution oriented, reflexive and oriented towards systems),^28^ reflecting in part limited input from allied health professionals. Our work builds on this existing literature, combining interviews and observation to provide a detailed analysis of how person‐centredness is enacted, how it is enacted by different professionals, and the barriers to its delivery.

Greenfield found a gap between the goals of integrated care initiatives, and the experience of many patients.^18^ Our work identified some of the ways in which practitioners could enact patient‐centredness in situations where the patient was not present, through sharing of information about the patient in meetings and capturing that information in the care records. Nevertheless, we did not identify many occurrences when knowledge about patient “wants, needs and preferences” was explicit in these discussions, meaning that the “patient as person” was generally filtered through the perspectives of professionals.

Harris found that discussions in MDTs often did not translate into actions.^28^ Our work suggests that when actions were suggested from meetings, there was still uncertainty as to whether the solutions would be acceptable to patients. One alternative observed in cancer MDT meetings shows decision making occurs “backstage,” with a consensus opinion or best treatment recommendations presented to patients “frontstage” in an MDT clinic.^26^ This provides an opportunity, albeit late, to be involved in decisions around their care. In work by O'Driscoll, patients with physical health problems seemed on the whole happy to defer decision making to “professionals” but liked having the opportunity to be involved if they wished.^29^

### 4.4 Implications for policy and practice

In the ICP, patient wishes and needs were not always known or taken into account. Greater involvement prior to, and after meetings (if not directly at them), could encourage better solutions, ensuring older people are signposted to the most relevant services and avoiding MDG recommendations that patients may not adopt. However, such high levels of patient involvement would likely slow implementation, clashing with the population approach to the MDGs, and the need to demonstrate rapid progress on integration due to wider service pressures.

Although the “Sally Ford” model encouraged staff to think what factors were likely to be important to people, this falls short of tailoring care to individual need. Although operationally the MDG protocol set out the pathway for consenting and consulting patients, the “Sally Ford” model might have inadvertently sent the message to staff that direct patient involvement was not necessary, limiting the person‐centredness achieved by the MDGs. Most reference to “Sally Ford” in interviews was around her as a “level” requiring certain types of care, not as a person at the centre of delivery.

Our findings show a genuine desire across all the staff roles involved in MDG meetings to engage with patients in the delivery of person‐centred care. Project leaders need to provide clear messages to maximize patient involvement, but the introduction of new models of care often have short timescales to realize results. A conceptual model of shared decision making which incorporates interprofessional working,^30^, ^31^ acknowledges that time and resource are likely barriers to potential implementation. Despite the need for “pace and scale” adoption of integrated care, those involved in reconfiguration of large teams need time to embed new practices.^32^

However, tensions between the aims of integrated and person‐centred care are likely to remain. It has been suggested that a person‐centred model of care for older people might be better achieved through alternative policy innovations, which might include personal health budgets^33^ or direct payments (allowing individuals to join up services in ways that make sense to them), rather than organizational and professional integration.^34^ The comparative advantages and disadvantages of these different approaches would be worthy of study, if the aims of integrated models to enhance patient experience are to be met.

## CONFLICT OF INTERESTS

There are no conflict of interests to declare.
